# From Scalp to Ear-EEG: A Generalizable Transfer Learning Model for Automatic Sleep Scoring in Older People

**DOI:** 10.1109/JTEHM.2024.3388852

**Published:** 2024-04-17

**Authors:** Ghena Hammour, Harry Davies, Giuseppe Atzori, Ciro Della Monica, Kiran K. G. Ravindran, Victoria Revell, Derk-Jan Dijk, Danilo P. Mandic

**Affiliations:** Department of Electrical and Electronic EngineeringImperial College London 4615 SW7 2BT London U.K.; 2Surrey Sleep Research Centre, School of Biosciences, Faculty of Health and Medical SciencesUniversity of Surrey 3660 GU2 7XH Guildford U.K.; U.K. Dementia Research Institute, Care Research and Technology Centre SW7 2BT London U.K.

**Keywords:** Automatic sleep scoring, hearables, ear-EEG, machine learning, wearable EEG

## Abstract

Objective: Sleep monitoring has extensively utilized electroencephalogram (EEG) data collected from the scalp, yielding very large data repositories and well-trained analysis models. Yet, this wealth of data is lacking for emerging, less intrusive modalities, such as ear-EEG.Methods and procedures: The current study seeks to harness the abundance of open-source scalp EEG datasets by applying models pre-trained on data, either directly or with minimal fine-tuning; this is achieved in the context of effective sleep analysis from ear-EEG data that was recorded using a single in-ear electrode, referenced to the ipsilateral mastoid, and developed in-house as described in our previous work. Unlike previous studies, our research uniquely focuses on an older cohort (17 subjects aged 65-83, mean age 71.8 years, some with health conditions), and employs LightGBM for transfer learning, diverging from previous deep learning approaches. Results: Results show that the initial accuracy of the pre-trained model on ear-EEG was 70.1%, but fine-tuning the model with ear-EEG data improved its classification accuracy to 73.7%. The fine-tuned model exhibited a statistically significant improvement (p < 0.05, dependent t-test) for 10 out of the 13 participants, as reflected by an enhanced average Cohen’s kappa score (a statistical measure of inter-rater agreement for categorical items) of 0.639, indicating a stronger agreement between automated and expert classifications of sleep stages. Comparative SHAP value analysis revealed a shift in feature importance for the N3 sleep stage, underscoring the effectiveness of the fine-tuning process.Conclusion: Our findings underscore the potential of fine-tuning pre-trained scalp EEG models on ear-EEG data to enhance classification accuracy, particularly within an older population and using feature-based methods for transfer learning. This approach presents a promising avenue for ear-EEG analysis in sleep studies, offering new insights into the applicability of transfer learning across different populations and computational techniques.Clinical impact: An enhanced ear-EEG method could be pivotal in remote monitoring settings, allowing for continuous, non-invasive sleep quality assessment in elderly patients with conditions like dementia or sleep apnea.

## I. Introduction

Sleep is an intrinsic physiological process which is vital for sustaining both cognitive function and overall well-being. Indeed, it plays a crucial role in numerous vital functions, for example by enhancing memory, boosting cognitive performance, and promoting dealing with stress [6]. On the other hand, sleep disturbances can have health implications, spanning from cognitive impairments to broader health concerns such as cardiovascular ailments, metabolic complications, heightened mortality risk, and recently identified ties to the progression of Alzheimer’s disease [7], [8], [9], [10]. As individuals sleep, their brain rhythms go through multiple stages of physiological and neurological restorative processes. Given its profound implications, there has been a growing interest to analyze human sleep across research, clinical, and consumer landscapes.

Polysomnography (PSG), the gold standard for sleep evaluation, refers to the analysis of sleep based on measurements from multiple physiological sensors. This includes Electroencephalography (EEG), which tracks the brain’s electrical activity to discern different sleep stages and patterns. Electrooculography (EOG), recording eye movements to identify the Rapid Eye Movement (REM) stage of sleep, is also usually part of the PSG setup. Furthermore, Electromyography (EMG) monitors muscle activities, crucial for detecting phases of sleep, especially REM. Respiratory effort sensors play a key role by measuring breathing patterns and detecting anomalies such as apneas or hypopneas. Finally, Oxygen Saturation (SpO2) monitoring is employed to assess blood oxygen levels, aiding in identifying periods of reduced oxygenation [11]. Each of these modalities contributes to a comprehensive understanding of the participant’s sleep architecture and quality, facilitating accurate diagnosis and treatment of sleep-related conditions like sleep apnea and insomnia [12], [13].

However, the challenges that impact the ease-of-use and accuracy of PSG include the need for overnight stay of patients in specialized labs, introducing the “first-night effect” due to unfamiliar surroundings and potentially biasing results [14]. Furthermore, the high costs associated with PSG limit accessibility, especially for those in remote areas or with mobility challenges. The unsuitability of PSG for long-term sleep tracking is also reflected in night-to-night sleep variability [15], making single-session data potentially unrepresentative. Also, the manual epoch-by-epoch scoring by sleep technicians, though guided by strict criteria, can be prone to inter-scorer variability [16], casting doubts on its consistent reliability and scalability.

The advancement of wearable technologies has paved the way for monitoring sleep at home, although these devices may not capture all the signals recorded through PSG. Among the signals that must be analyzed for sleep scoring is at least the EEG signal. In this context, Hearables [17], devices worn in the ear, have emerged as a practical solution for sleep analysis [18], [19], [20], [21], [22]. Such devices can monitor various physiological and non-physiological signals including EEG [23], [24], electrocardiogram (ECG) [25], [26], [27], cognitive workload [28], and daily activities [29]. Findings from studies utilizing standardized ear-EEG sensors reveal a considerable correlation between automatic sleep stage prediction using ear-EEG and the hypnogram derived from a PSG [18], [19].

Hearables represent an emergent modality, so that large-scale datasets and automated sleep-staging models specific to ear-EEG are still in their infancy. On the other hand, sleep research has been an active area for many years and there is a wealth of publicly available databases of both scalp EEG datasets and automated sleep-staging models [2], [30], [31], [32]. These open-source models have been trained on data recorded from sleep labs worldwide, encompassing a wide range of demographics and sleep-related conditions. A natural question to be asked is: Can these open-source models, trained and optimized on scalp EEG, be employed effectively for ear-EEG data? To generalize well across different datasets and conditions, machine learning models typically require large amounts of data to avoid overfitting and to capture the diversity inherent in real-world applications. In-ear EEG, with its unique sensor technology, placement, and potential variations in signal quality, may have distinct characteristics from traditional scalp EEG. Successfully adapting these pre-trained models to ear-EEG could dramatically accelerate and facilitate the development of Hearables, optimizing both time and resources, a subject of this study.

Open-source models for automatic sleep staging can be broadly classified into two major categories: deep-learning-based and feature-based. Deep-learning based models include U-Sleep by Perslev et al. [31], SeqSleepNet from Phan et al. [2], and TinySleepNet by Supratak and Guo [30]. Each of these models, while architecturally distinct, predominantly trains on raw single-channel EEG data, with U-Sleep also integrating raw EOG data. The feature-based models include those developed by Vallat and Walker [32] who trained a LightGBM model, called YASA, on more than 30,000 hr of PSG data across 3163 full-night PSG recordings from a heterogeneous population. They achieved kappa values of more than 0.80, indicating a level of agreement that is higher than the inter-scorer agreement reported in literature. Contrasting the deep-learning counterparts, feature-based models use predefined features, encompassing statistical metrics of the signal (e.g. mean, variance, and skewness), spectral attributes (e.g. power within certain frequency bands), and other domain-specific features [19], [33]. Although transfer learning has been extensively demonstrated for deep learning models in ear-EEG analysis [2], [3], [4], [5], its application to feature-based models - such as LightGBM - remains unexplored, representing a gap that is addressed by the present study. In this study, we examine the extent to which the open-source YASA model can be adapted to ear-EEG data for sleep scoring through transfer learning. Our motivation is to leverage the considerable knowledge gained from the analysis of scalp EEG to improve performance on the ear-EEG task. It is important to mention that, unlike deep-learning models, feature-based models like LightGBM offer inherent interpretability and explainability, which is crucial for identifying both similarities and discrepancies when applying a model trained on scalp EEG to ear-EEG data.

## II. Methodology

### A. Participant Recruitment and Data Collection

A cohort of 17 participants (mean age: $71.8~\pm ~4.4$ years; age range: 65–83 years; 6 females) was enrolled in the study. All participants had no documented history of neurological or mental health issues. While some individuals reported comorbidities such as type-2 diabetes, sleep apnea, and hypertension, these conditions were stable, well-managed, and had not necessitated recent medication adjustments or hospital admissions.

**FIGURE 1.**
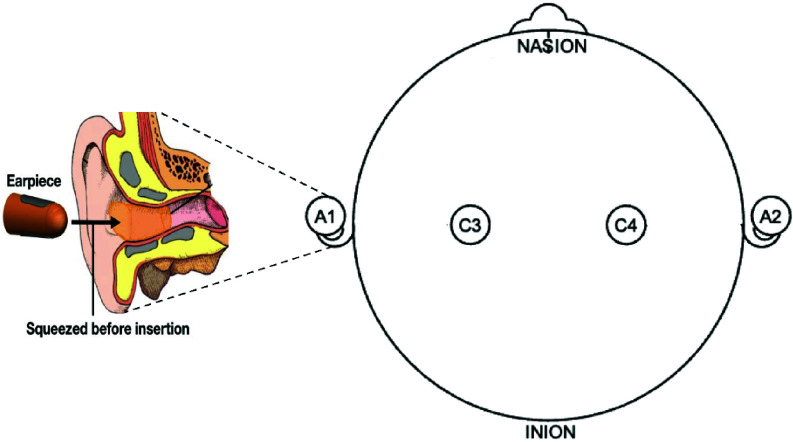
The experimental EEG setup, with the scalp electrodes and the generic Ear-EEG sensor visible.

The participants were invited for an overnight stay at the Surrey Sleep Research Centre (SSRC). During their stay, a comprehensive 10-hour in-bed polysomnography (PSG) assessment was conducted, aligning with the guidelines set by the American Academy of Sleep Medicine (AASM). The recordings implemented a standard AASM adult PSG montage, and concurrently, EEG was recorded from one ear using a viscoelastic generic earplug described in our previous work [1], [17] that conforms to the shape of the ear canal, with a flexible electrode fixed onto its surface shown in Figure 1. The choice between referencing the ear sensor to M1 or M2 was determined by the side of ear-EEG recording; the sensing electrode was placed inside the canal of one ear, with the reference electrode positioned on the ipsilateral mastoid (M1 for the left ear, M2 for the right ear). A SomnoHD recording system combined with the DOMINO software (Somnomedics, Germany) was employed for all scalp EEG and ear-EEG recordings. Sleep stages were divided into 30-second epochs and were independently reviewed by two scorers. Each scorer independently analyzed the EEG recordings to assign sleep stages according to standard criteria. The consensus hypnogram was then generated by comparing the scorers’ classifications, with discrepancies resolved through a joint review session to reach agreement [34].

All procedures in this study were executed adhering to the Declaration of Helsinki and Good Clinical Practice standards. The research design was granted approval by the NHS ethics committee (22/LO/0694). Prior to any study-related procedures, participants were briefed in detail about the research activities, and they gave their written informed consent.

### B. Data Pre-Processing

For every recorded subject, a central scalp EEG channel was extracted (C4-M1 or C3-M2) along with an ear-EEG channel referenced ipsilaterally to either M1 or M2, depending on which had the sensor. Out of the 17 recorded participants, 4 were removed from this study due to the high impedance (i.e. higher than 10k Ohm) between the skin and the electrode which heavily impaired signal quality. High impedance was noticed whenever the subject refused to clean their ear before the insertion of the sensors. Impedance measurements were conducted before sleep recordings to ensure signal quality; however, due to technical constraints, continuous impedance monitoring throughout the night was not feasible, limiting our ability to assess the impact of impedance variations on staging accuracy. The included signals were then downsampled from 256 Hz to 100 Hz and bandpass-filtered between 0.40 Hz and 30 Hz, to match the lightGBM model that scalp EEG data had been trained on. Features were then calculated based on 30-sec epochs to train the machine learning model.

### C. Features

The procedure for extracting features from EEG signals integrates both time- and frequency-domain analysis. In the time domain, common descriptive metrics such as the standard deviation, skewness, interquartile range, and kurtosis were employed. Furthermore, non-linear characteristics like the count of zero-crossings, Hjorth mobility and complexity parameters, permutation entropy, and the fractal dimension of the signal were determined. In the frequency domain, the periodogram, computed for each 30-second epoch using Welch’s approach, serves as the basis for deriving features, encompassing specific band relative spectral powers, the broadband signal’s absolute power, and power ratios. The full set of features is given in Table 1.

**TABLE 1:** List of Features Computed From EEG Data for Sleep Classification [32]

Feature name	Description
Absolute power	Total energy across all frequencies Total energy across all frequencies
Absolute power	Total energy across all frequencies
Alpha power	Energy in the alpha frequency band (8-13 Hz)
Alpha/theta power ratio	Proportional energy between alpha and theta bands
Beta power	Energy in the beta frequency band (13-30 Hz)
Delta/beta power ratio	Proportional energy between delta and beta bands
Delta/sigma power ratio	Proportional energy between delta and sigma bands
Delta/theta power ratio	Proportional energy between delta and theta bands
Fast delta power	Energy in the upper range of delta frequency band
Hjorth complexity	Measure of signal complexity compared to its derivatives
Higuchi fractal dimension	Quantifies the complexity of time series
Hjorth mobility	Rate of change of signal amplitude
Time elapsed (hours)	Duration from the start of EEG recording
Inter-quartile range	Range between 25th and 75th percentiles of data distribution
Kurtosis	Measure of the data’s peakedness or flatness relative to normal distribution
Time elapsed (normalized)	Standardized duration from start of EEG recording
Number of zero-crossings	Times the signal crosses the zero amplitude
Permutation entropy	Uncertainty measure in the order of amplitude values
Petrosian fractal dimension	Quantifies the roughness or irregularity of signals
Slow delta power	Energy in the lower range of delta frequency band
Sigma power	Energy in the sigma frequency band (typically related to sleep spindles)
Skewness	Asymmetry in the data distribution
Standard deviation	Dispersion or variability of the signal
Theta power	Energy in the theta frequency band (4-8 Hz)

To capture the temporal dynamics inherent in sleep EEG data, each feature was additionally smoothed in two distinct manners: using a 2-minute rolling window or, alternatively, using a 7.5-minute triangular window. Incorporating these dual variants enabled the model to assimilate information from adjacent epochs. Every smoothed feature was then normalized across each night. The model uses as input both smoothed and raw features in the original units. All features were calculated using the YASA sleep analysis toolbox in Python [32].

### D. Mutual Information Analysis

Mutual Information (MI) is an information-theoretic measure that quantifies the dependency between two random variables. In the context of our study, we employed MI to quantify the relationship between features extracted from EEG signals and the corresponding sleep stages ([35]). For each participant, features were calculated from both ear and scalp EEG data. Each feature’s value, for every epoch, was paired with the corresponding sleep stage, resulting in a two-dimensional dataset. The MI between EEG features and sleep stages was computed as

\begin{equation*} MI(X,Y) = \sum \limits _{y \in Y} \sum \limits _{x \in X} p(x,y) \log \left ({{ \frac {p(x,y)}{p(x)p(y)} }}\right )\end{equation*}

where *X* represents the EEG features, *Y* represents the sleep stages, $p(x,y)$ is the joint probability distribution function of *X* and *Y*, and $p(x)$ and $p(y)$ are the marginal probability distribution functions of *X* and *Y*, respectively.

All MI calculations and visualizations were performed in Python, using the Scipy library for MI computations and Matplotlib for visualizations.

### E. Pre-Trained Model

The open-source pre-trained model from [32] was built upon a comprehensive training set encompassing over 31,000 hours of PSG data derived from 3,163 distinct recordings distributed across seven datasets. The scoring for this vast dataset was achieved through a consensus of five sleep technicians, ensuring its reliability and representing a considerable breadth of real-world sleep data. The core of the model is a LightGBM classifier [36], a gradient-boosting framework which uses tree-based algorithms. This classifier was tuned with specific hyper-parameters: 500 estimators, a tree depth capped at 5, up to 90 leaves per tree, and it utilized 60% of the available features for the construction of each tree. These hyper-parameters were carefully chosen from 96 potential combinations to deter overfitting while optimizing accuracy, achieved through an exhaustive threefold cross-validation on the entire training set. Notably, to address the inherent imbalance in the distribution of sleep stages during a typical night, custom weights were applied to the sleep stages. After rigorous optimization, the chosen weights were 2.2 for N1, 1 for both N2 and Wake stages, 1.2 for N3, and 1.4 for REM. The model, once trained, was exported as a compact file and made available from https://github.com/raphaelvallat/YASA.

### F. Fine-Tuned Model

The open-source pre-trained LightGBM model was adapted for our specific datasets using fine-tuning. Contrary to traditional retraining or adaptation approaches, fine-tuning, in this context, does not involve altering the hyperparameters or modifying the existing decision trees that the original model has learned. Instead, it can be viewed as an expansion of the pre-existing model. Fine-tuning was implemented by passing the pre-trained model as the argument for the “init model” parameter when training a LightGBM model on new data. The hyperparameters of the pre-trained model were not modified.

Upon introducing our new dataset into the model, rather than restructuring or pruning the current decision trees, new decision trees were constructed and appended based on the patterns and information obtained from our data [36]. This ensures that the model retains the knowledge and insights it gained from the vast dataset on which it was originally trained while simultaneously integrating the nuances of our specific ear-EEG or scalp EEG datasets. This fine-tuning approach capitalizes on the inherent strengths of gradient-boosting frameworks, where new trees are added iteratively, minimizing the residual errors from prior trees. The result is a harmonious blend of generalized knowledge from the pre-trained model with specific insights from the new scalp or ear-EEG data. Due to the small size of the datasets used for fine-tuning, the sum of the weights of the newly-added trees amounted to only 0.15% of that of the pre-trained model.

For this study, the pre-trained LightGBM model underwent a separate fine-tuning process for each dataset: one for the ear-EEG data and another for the scalp EEG data. The model was fine-tuned using the LightGBM method for continued training, and the fine-tuned model was evaluated using cross-validation across participants. The pre-trained LightGBM model was fine-tuned using the scalp or in-ear data of all participants except for one, and the fine-tuned model was used to predict the sleep stages of the subject that had been left out, iterating over all participants in the dataset.

### G. Shap Analysis

SHapley Additive exPlanations (SHAP) is a game theoretic approach to explain the output of any machine learning model. In this study, SHAP values were utilized to interpret the contributions of individual features to the predictions made by both the pre-trained and fine-tuned models for ear-EEG signals [37]. For every prediction made by the model, a SHAP value was computed for each feature. The SHAP value represents the average contribution of the given feature to every possible prediction. It is computed based on Shapley values from cooperative game theory. Given a prediction model, *f*, for an instance *x* and a feature *i*, the SHAP value, $shap_{i}(x)$, is calculated as

\begin{align*} & shap_{i}(x) \\ & = \sum _{S \subseteq F \setminus \{i\}} \frac {|S|!(|F|-|S|-1)!}{|F|!} [f_{S}(x_{S} \cup \{x_{i}\}) - f_{S}(x_{S})]\end{align*}

where *F* is the set of all features and *S* is a subset of *F*.

Higher magnitude SHAP values indicate features with more influence on the model’s prediction for a specific instance. By analyzing the top contributing features, we could determine the key EEG features that the models relied upon for sleep stage classification, and observe any shifts in feature importance after model fine-tuning. The SHAP analysis and subsequent visualizations were conducted using the SHAP Python library. This library offers efficient algorithms to estimate SHAP values for a wide range of models and provides utilities for visualizing the results.

## III. Results

**FIGURE 2.**
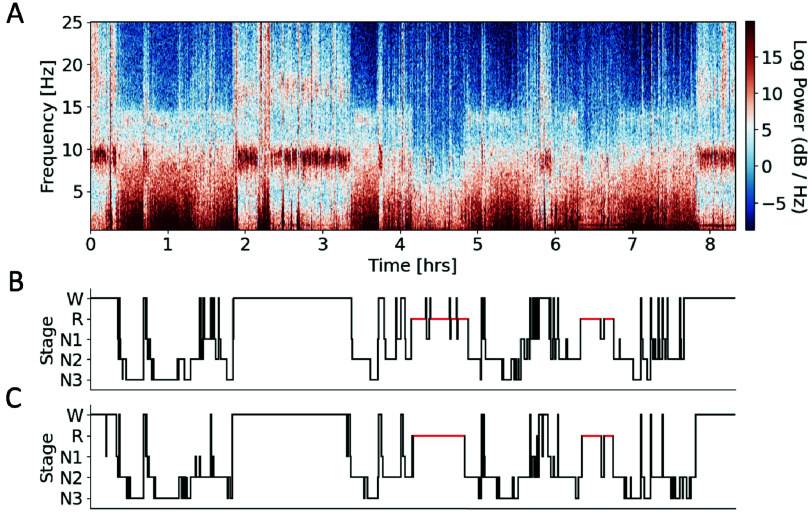
Sleep data for a representative participant. (A) Time-frequency spectrogram of ear-EEG recorded during one night of sleep, showing the frequency distribution over time. (B) Consensus hypnogram, illustrating the ground-truth sleep stages throughout the night. Red lines indicate periods of REM sleep. (C) Predicted hypnogram using the ear-EEG, generated by the pre-trained model.

Both the ear and scalp electroencephalogram (EEG) were recorded during a full-night sleep from a select group of older adults (n =13, Methods II-A). Figure 2 shows data collected from one representative participant including the time-frequency spectrogram from the ear-EEG, and the consensus hypnogram (a sleep expert’s manually annotated representation of the sleep cycle). Using these data, the sleep stages were automatically estimated with an open-source LightGBM model pre-trained on a very large scalp EEG dataset, and the resulting hypnogram was generated for the ear-EEG (Fig. 2C). The same analysis was conducted for the scalp EEG recordings.

**FIGURE 3.**
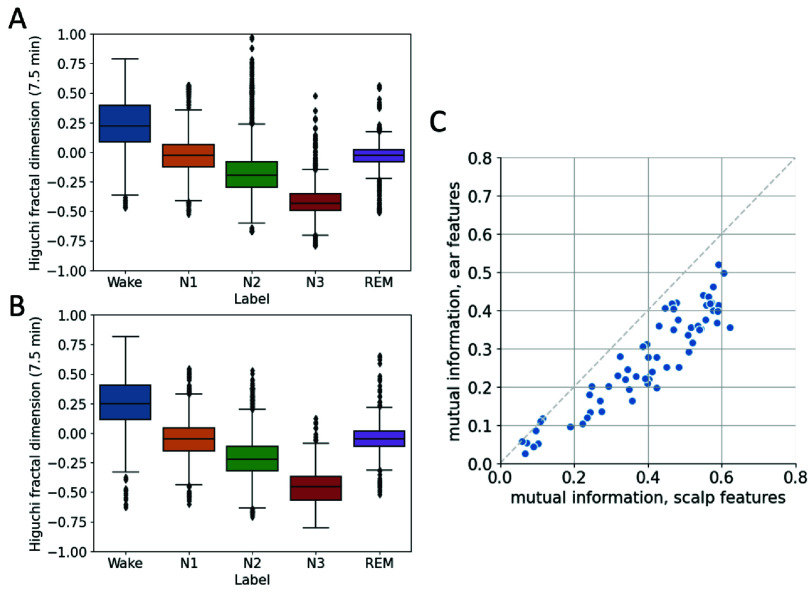
Comparative analysis of the Higuchi fractal dimension feature across EEG modalities and its relation to sleep stages. (A) Boxplot distributions showcasing the spread of the Higuchi fractal dimension feature derived from ear-EEG across all sleep stages and participants. (B) Corresponding boxplot distributions for the same feature, but derived from scalp EEG data. (C) Scatterplot visualizing mutual information scores between feature values and sleep stages, with higher scores indicating greater similarity between features and sleep stages. Features from scalp EEG (x-axis) had higher mutual information with sleep stages than features from ear-EEG (y-axis).

A comprehensive set of 65 distinctive features was extracted from each 30-second epoch of both the in-ear and scalp EEG channels (Methods II-C). Figure 3 illustrates the distributions for one of the features (Higuchi fractal dimension) calculated from both in-ear and scalp EEG across all sleep stages and participants. Initial visual inspection of these distributions suggested a slight advantage for scalp EEG (ANOVA F =6900.3, p < 0.01), displaying less overlap across sleep stages compared to ear-EEG (ANOVA F =4530.3, p < 0.01). This visual inference was confirmed quantitatively by computing the mutual information—an information-theoretic measure of the dependence between the features and the sleep stages (Methods II-D). When comparing mutual information scores, 62 out of 65 features derived from scalp EEG demonstrated higher values than their ear-EEG counterparts (Fig. 3C). The results point to a relatively higher delineation of sleep stages using scalp EEG features, although the ear-EEG data also offers a significant level of differentiation.

**FIGURE 4.**
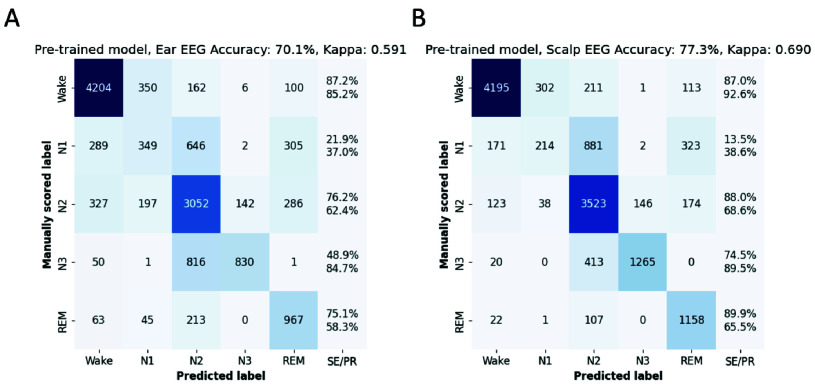
Comparison of sleep stage predictions by the pre-trained model across EEG modalities. (**A**) Confusion matrix illustrating the classification results of the pre-trained model using ear-EEG data over all sleep epochs across participants. (**B**) Confusion matrix, but based on scalp EEG data, highlighting the performance differences between the two modalities. SE/PR denote respectively sensitivity (above) and precision (below).

The accuracy of sleep stage classification utilizing the pre-trained LightGBM classifier on both in-ear and scalp EEG features is visualized in Figure 4. Overall, scalp EEG offered a higher accuracy rate of 77.3%, in comparison to 70.1% accuracy achieved by ear-EEG. In terms of sensitivity, the largest difference between the two modalities was observed in the N3 stage, where scalp EEG achieved a notably higher sensitivity of 77.8% compared to the ear-EEG sensitivity of 48.9%. A more detailed account of the results for each participant is provided in Table 2. Here, it was observed that scalp EEG yielded higher kappa scores—indicating superior model performance—for 10 out of the 13 participants and averaged a kappa score of 0.685. In contrast, the ear-EEG yielded a lower average kappa score of 0.589.

**TABLE 2:** Sleep Analysis Results for All Participants (pre: Pre-Trained Model; Fine: Fine-Tuned Model

Gender	Cohen’s Kappa				Accuracy			
	Scalp (pre)	Scalp (fine)	In-ear (pre)	In-ear (fine)	Scalp (pre)	Scalp (fine)	In-ear (pre)	In-ear (fine)
Female	0.758	0.693	0.687	0.750	0.824	0.778	0.771	0.819
Male	0.673	0.678	0.550	0.642	0.749	0.756	0.666	0.734
Male	0.641	0.680	0.608	0.682	0.741	0.775	0.717	0.766
Male	0.577	0.553	0.578	0.603	0.695	0.673	0.700	0.721
Male	0.699	0.659	0.546	0.547	0.792	0.756	0.686	0.683
Male	0.623	0.701	0.587	0.623	0.724	0.779	0.700	0.722
Male	0.719	0.702	0.513	0.652	0.793	0.777	0.643	0.736
Female	0.671	0.641	0.545	0.517	0.763	0.735	0.665	0.643
Female	0.723	0.746	0.467	0.679	0.795	0.805	0.602	0.757
Male	0.623	0.622	0.651	0.626	0.740	0.729	0.765	0.740
Male	0.735	0.743	0.584	0.586	0.802	0.806	0.689	0.687
Female	0.792	0.729	0.792	0.778	0.848	0.800	0.848	0.836
Male	0.664	0.705	0.542	0.628	0.765	0.792	0.669	0.740

**FIGURE 5.**
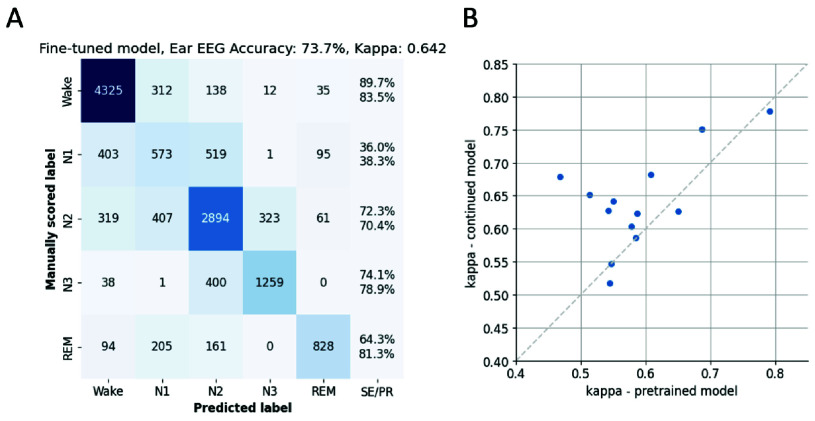
Enhanced sleep stage classification results following continued training on the ear-EEG dataset. (A) Confusion matrix depicting the classification outcomes of the fine-tuned model using ear-EEG data over all sleep epochs across all participants, showcasing improved accuracy and sensitivity. SE/PR denote respectively sensitivity (above) and precision (below). (B) Scatterplot presenting a direct comparison of kappa scores per subject: those derived from the pre-trained model on the x-axis versus the kappa scores from the fine-tuned model on the y-axis, highlighting the enhancement in model performance post fine-tuning.

**FIGURE 6.**
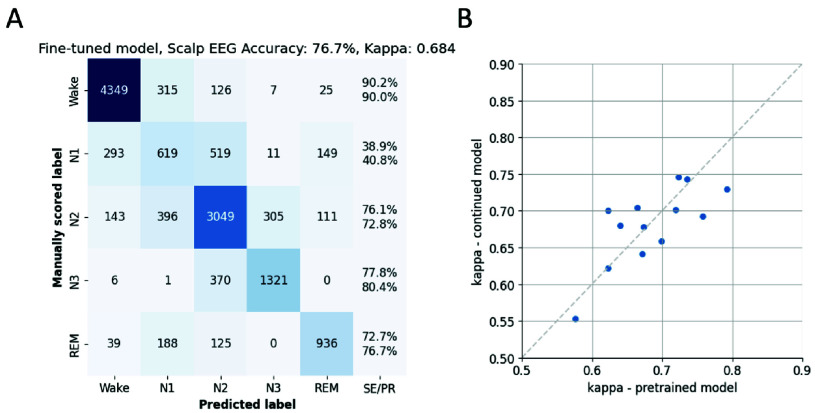
Results of continued training on the scalp EEG dataset, illustrating the contrasting effect compared to ear-EEG fine-tuning. (A) Confusion matrix presenting the classification outcomes of the fine-tuned model using scalp EEG data over all sleep epochs across participants, underscoring the limited improvement in performance. SE/PR denote respectively sensitivity (above) and precision (below). (B) Scatterplot offering a direct juxtaposition of kappa scores: values derived from the pre-trained model on the x-axis against those from the fine-tuned model on the y-axis, indicating the minimal variance in model efficacy despite the continued training approach.

The pre-trained LightGBM model was fine-tuned by continued training using the present in-ear and scalp EEG datasets (Methods II-F). This procedure aimed to optimize the model’s performance by fine-tuning it on the specific characteristics of ear-EEG with respect to sleep stages. The impact of this refinement process was evaluated via cross-validation across all participants. Figure 5 displays the results of the continued training using the in-ear dataset. The confusion matrix (Fig. 5A) reveals a marked improvement in the performance of the model, particularly in the sensitivity for N3, which rose from 48.9% to 74.1%. Additionally, the overall accuracy increased to 73.7%. The model fine-tuned on ear-EEG demonstrated increased kappa values for 10 out of the 13 participants (Fig. 5B), yielding an overall kappa score of 0.639, which constitutes a statistically significant improvement over the performance of the pre-trained model (p < 0.05, dependent t-test). Importantly, the continued training strategy did not yield similar improvements for scalp EEG. As demonstrated in Figure 6, the fine-tuned model on scalp EEG data yielded similar accuracy (Fig. 6A; average accuracy: 76.7%) and kappa values (Fig. 6B; overall kappa-score: 0.681) as those observed in the pre-trained model, suggesting a limit to the improvement possible with this strategy on our scalp EEG dataset.

**FIGURE 7.**
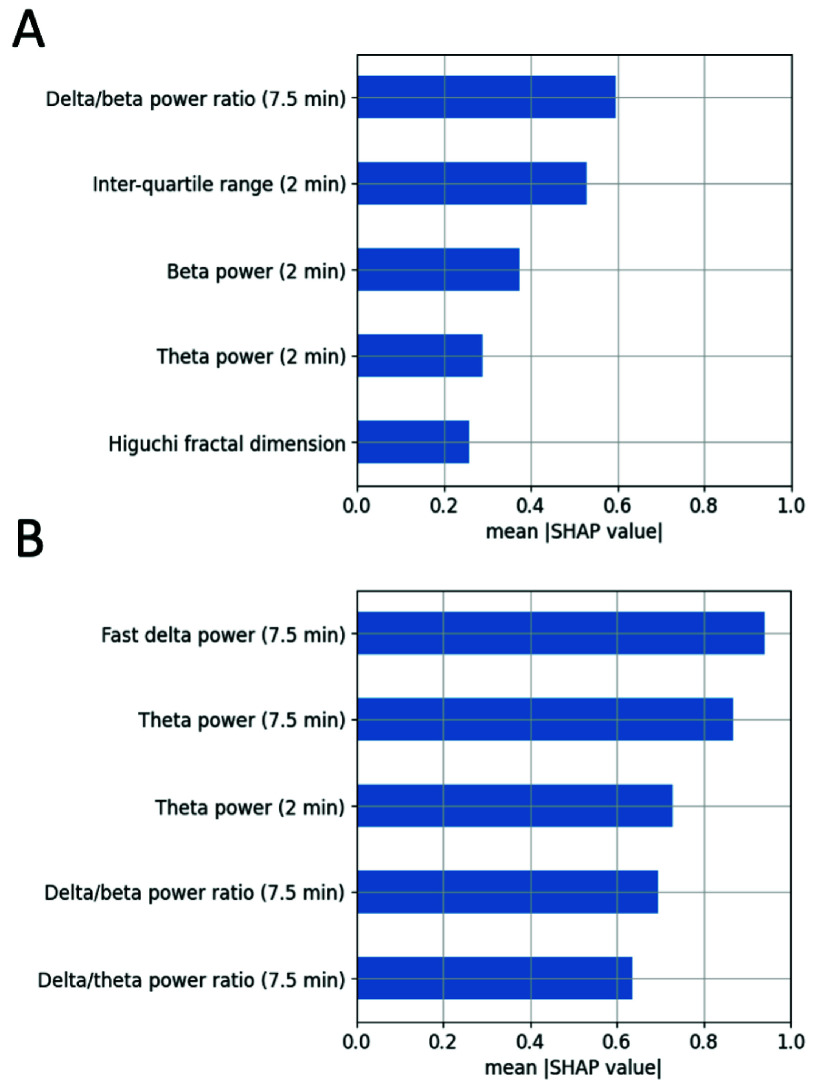
Comparative analysis of the highest average SHAP values associated with predicting the N3 sleep stage using ear-EEG features. (A) Bar chart depicting the top 5 SHAP values and their corresponding features as determined by the pre-trained model, offering insights into the most influential features before fine-tuning. (B) Corresponding bar chart for the top 5 SHAP values and their associated features for the fine-tuned model, highlighting the shift in feature importance and relevance post model refinement.

The SHAP analysis, an approach used for interpreting machine learning models, was next conducted on the ear-EEG dataset (Methods II-G). The results are depicted in Figure 7, which demonstrates the highest average SHAP values for predicting the N3 sleep stage during N3 epochs, comparing the pre-trained model (Fig. 7A) and the fine-tuned model (Fig. 7B). A notable difference in the two models can be observed in the significance attributed to different EEG features. The fast delta power, an EEG feature closely associated with the N3 sleep stage, had the highest SHAP value in the fine-tuned model. However, this feature did not appear among the top five most influential features in the pre-trained model. Similarly, the beta power—strongly linked to awake brain activity—had the third highest SHAP value in the pre-trained model but was not among the top five in the fine-tuned model. These results highlight how continued training reconfigures the importance of various EEG features for sleep stage classification, enhancing the model’s precision in predicting the N3 stage based on ear-EEG data.

## IV. Discussion

This study aimed to examine the efficacy of automatic sleep staging in older adult participants using ear-EEG signals, by leveraging an open-source model pre-trained on a large scalp EEG dataset. The results demonstrated substantial agreement between the sleep stages predicted from in-ear and scalp EEG, suggesting a high degree of similarity between these two modalities. The overall performance of the model, as quantified by kappa scores, showed consistency across both types of EEG inputs, though it was slightly higher for the scalp EEG. This supports previous research suggesting that despite the varying locations and spatial resolutions, in-ear and scalp EEG can capture similar neuronal activity patterns essential for sleep stage identification.

One intriguing observation from the present analysis was the higher mutual information of scalp EEG with sleep stages as compared to ear-EEG. This result is, to some extent, intuitive given the closer proximity of scalp electrodes to cortical processes that generate the slow EEG waveforms typically associated with sleep. Such waveforms include delta and theta waves which are crucial for defining certain sleep stages. Previous studies have emphasized the importance of electrode location in achieving accurate sleep staging, with optimal locations often being directly on the scalp [38]. These superior feature metrics naturally favored scalp EEG, leading to its enhanced performance for sleep stage classification compared to ear-EEG. However, it is worth noting that, despite its relative disadvantage in terms of feature salience, ear-EEG demonstrated a reasonably high level of accuracy and sensitivity.

The fine-tuning of the pre-trained model led to significant enhancements in sleep stage prediction from ear-EEG data, most notably for the N3 sleep stage. The initial underperformance of the pre-trained model in detecting the N3 sleep stage from ear-EEG could be attributed to the distinct patterns of neuronal activity that define this stage of sleep, often referred to as slow-wave sleep. Given the inherent differences in spatial resolution and proximity to the brain between scalp and ear-EEG, it is plausible that the pre-trained model, trained largely on scalp EEG data, was not fully equipped to recognize the unique manifestations of N3 sleep stage in the ear-EEG data.

However, through continued training and refinement with the specific ear-EEG dataset, the model was effectively fine-tuned to better recognize and interpret these distinctive signal patterns. Particularly interesting was the rising importance of fast delta power— a feature strongly associated with N3 sleep stage— in the fine-tuned model. This feature, which was not among the top influencers in the pre-trained model, emerged as the most influential feature in the fine-tuned model, underscoring the adaptive nature of the machine learning algorithm and its ability to optimize its predictive capabilities according to the input data. This shift in influential features after fine-tuning also illustrates the importance of domain-specific knowledge and model personalization in the development of accurate sleep stage classifiers.

In our study, the pre-trained LightGBM model attained an average kappa score of 0.685 on single-channel scalp EEG data from our cohort. This figure contrasts with findings from Vallat and Walker [32], who reported a higher median kappa score of 0.782 using the same model on single-channel EEG data. They also identified a significant negative correlation between model accuracy and both the age of subjects and the severity of sleep apnea symptoms. They suggested that diminished model performance might be attributed to the increased frequency of stage transitions common in individuals with sleep apnea. Given these insights, the lower performance observed in our dataset can likely be attributed to the demographic characteristics of our cohort, which has an average age of 71.8 years, significantly older than the 45.3 years average age of the cohort in Vallat and Walker’s study [32]. Additionally, the potentially higher prevalence of sleep apnea within our older cohort could further explain the reduced kappa score. This divergence underscores the need to consider the impact of demographic and clinical variables on model performance in sleep study analyses, which may have implications for the generalizability of findings across different populations. Due to the small sample size of our dataset, comprising 13 subjects, the results obtained are indicative but not definitive, necessitating further validation through larger-scale studies in the future.

In a broader perspective, these results mark a significant advancement towards the realization of user-friendly, home-based sleep monitoring solutions, particularly beneficial for the estimated 50% of older adults who suffer from sleep disorders [39]. The advantages of ear-EEG, with its non-invasive nature, compact size, and reduced susceptibility to artifacts compared to traditional scalp EEG, not only make it well-suited for use in various clinical and research settings but also offer a promising avenue for continuous, long-term sleep monitoring in the community. This approach could significantly enhance our capacity for personalized sleep research and treatment strategies, offering a tailored approach to managing and mitigating sleep disorders in the ageing population, thereby improving their overall quality of life and health outcomes.

## V. Conclusion

The present findings have illuminated the potential utility of ear-EEG as a viable, minimally invasive technique for sleep stage classification, particularly in older populations where comfort and ease of use are critical factors. The robust performance of the pre-trained model on the ear-EEG data — further optimized through fine-tuning — has provided compelling evidence for the adaptability and practicality of this modality. The significant improvement in N3 sleep stage classification achieved through the fine-tuning process has indicated that, with model personalization, the potential barriers associated with the use of ear-EEG for sleep staging can be effectively addressed. Moreover, the shift in influential EEG features post fine-tuning further underscores the ability of the model to adapt to unique data characteristics and optimize its predictive power.

Our study has introduced the application of ear-EEG for sleep analysis within an older population, a group often overlooked in sleep research yet significantly affected by sleep disorders. This approach not only explores new ground but also suggests potential for developing accessible, non-invasive monitoring tools that could improve quality of life. Additionally, opting for LightGBM over the previously used deep learning frameworks for transfer learning [2], [3], [5] provides a new feature-based method. LightGBM’s advantages in terms of explainability, lower computational needs, and ease of use contribute to its potential for fostering quicker advancements and applications in the field of sleep study, making it a valuable tool for future research and development in sleep monitoring technologies.

## References

[1] V. Goverdovsky, D. Looney, P. Kidmose, and D. P. Mandic, “In-ear EEG from viscoelastic generic earpieces: Robust and unobtrusive 24/7 monitoring,” IEEE Sensors J., vol. 16, no. 1, pp. 271–277, Jan. 2016.

[2] H. Phan, F. Andreotti, N. Cooray, O. Y. Chén, and M. De Vos, “SeqSleepNet: End-to-end hierarchical recurrent neural network for sequence-to-sequence automatic sleep staging,” IEEE Trans. Neural Syst. Rehabil. Eng., vol. 27, no. 3, pp. 400–410, Mar. 2019. 30716040 10.1109/TNSRE.2019.2896659 PMC6481557

[3] H. Phan , “L-SeqSleepNet: Whole-cycle long sequence modeling for automatic sleep staging,” IEEE J. Biomed. Health Informat., vol. 27, no. 10, pp. 4748–4757, Oct. 2023. 10.1109/JBHI.2023.3303197 37552591

[4] H. Phan, O. Y. Chén, P. Koch, A. Mertins, and M. D. Vos, “Deep transfer learning for single-channel automatic sleep staging with channel mismatch,” in Proc. 27th Eur. Signal Process. Conf. (EUSIPCO), Sep. 2019, pp. 1–5.

[5] K. B. Mikkelsen, H. Phan, M. L. Rank, M. C. Hemmsen, M. de Vos, and P. Kidmose, “Sleep monitoring using ear-centered setups: Investigating the influence from electrode configurations,” IEEE Trans. Biomed. Eng., vol. 69, no. 5, pp. 1564–1572, May 2022. 34587000 10.1109/TBME.2021.3116274

[6] M. P. Walker, “The role of sleep in cognition and emotion,” Ann. New York Acad. Sci., vol. 1156, no. 1, pp. 168–197, 2009. 19338508 10.1111/j.1749-6632.2009.04416.x

[7] F. P. Cappuccio and M. A. Miller, “Sleep and cardio-metabolic disease,” Current Cardiol. Rep., vol. 19, no. 11, pp. 1–9, Nov. 2017. 10.1007/s11886-017-0916-0 PMC5605599 28929340

[8] L. Besedovsky, T. Lange, and M. Haack, “The sleep-immune crosstalk in health and disease,” Physiological Rev., vol. 99, no. 3, pp. 1325–1380, Jul. 2019. 10.1152/physrev.00010.2018 PMC6689741 30920354

[9] F. P. Cappuccio, L. D’Elia, P. Strazzullo, and M. A. Miller, “Sleep duration and all-cause mortality: A systematic review and meta-analysis of prospective studies,” Sleep, vol. 33, no. 5, pp. 585–592, May 2010. 20469800 10.1093/sleep/33.5.585 PMC2864873

[10] J. R. Winer , “Sleep disturbance forecasts β-amyloid accumulation across subsequent years,” Current Biol., vol. 30, no. 21, pp. 4291–4298, Nov. 2020. 10.1016/j.cub.2020.08.017 PMC7642104 32888482

[11] J. V. Rundo and R. Downey, “Chapter 25—Polysomnography,” in Clinical Neurophysiology: Basis and Technical Aspects, vol. 160, K. H. Levin and P. Chauvel, Eds. Amsterdam, The Netherlands: Elsevier, 2019, pp. 381–392. 10.1016/B978-0-444-64032-1.00025-4 31277862

[12] V. K. Kapur , “Clinical practice guideline for diagnostic testing for adult obstructive sleep apnea: An American academy of sleep medicine clinical practice guideline,” J. Clin. Sleep Med., vol. 13, no. 3, pp. 479–504, Mar. 2017. 28162150 10.5664/jcsm.6506 PMC5337595

[13] T. Andrillon , “Revisiting the value of polysomnographic data in insomnia: More than meets the eye,” Sleep Med., vol. 66, pp. 184–200, Feb. 2020. 31978862 10.1016/j.sleep.2019.12.002

[14] J.-H. Byun, K. T. Kim, H.-J. Moon, G. K. Motamedi, and Y. W. Cho, “The first night effect during polysomnography, and patients’ estimates of sleep quality,” Psychiatry Res., vol. 274, pp. 27–29, Apr. 2019. 30776709 10.1016/j.psychres.2019.02.011

[15] L. Fiorillo , “Automated sleep scoring: A review of the latest approaches,” Sleep Med. Rev., vol. 48, Dec. 2019, Art. no. 101204. 10.1016/j.smrv.2019.07.007 31491655

[16] R. S. Rosenberg and S. Van Hout, “The American academy of sleep medicine inter-scorer reliability program: Sleep stage scoring,” J. Clin. Sleep Med., vol. 9, no. 1, pp. 81–87, Jan. 2013. 23319910 10.5664/jcsm.2350 PMC3525994

[17] V. Goverdovsky , “Hearables: Multimodal physiological in-ear sensing,” Sci. Rep., vol. 7, no. 1, p. 6948, Jul. 2017. 28761162 10.1038/s41598-017-06925-2 PMC5537365

[18] T. Nakamura, Y. D. Alqurashi, M. J. Morrell, and D. P. Mandic, “Hearables: Automatic overnight sleep monitoring with standardized in-ear EEG sensor,” IEEE Trans. Biomed. Eng., vol. 67, no. 1, pp. 203–212, Jan. 2020. 31021747 10.1109/TBME.2019.2911423

[19] T. Nakamura, V. Goverdovsky, M. J. Morrell, and D. P. Mandic, “Automatic sleep monitoring using ear-EEG,” IEEE J. Transl. Eng. Health Med., vol. 5, pp. 1–8, 2017. 10.1109/JTEHM.2017.2702558 PMC5515509 29018638

[20] D. Looney, V. Goverdovsky, I. Rosenzweig, M. J. Morrell, and D. P. Mandic, “Wearable in-ear encephalography sensor for monitoring sleep. Preliminary observations from nap studies,” Ann. Amer. Thoracic Soc., vol. 13, no. 12, pp. 2229–2233, Dec. 2016. 10.1513/AnnalsATS.201605-342BC PMC5291497 27684316

[21] Y. R. Tabar, K. B. Mikkelsen, M. L. Rank, M. C. Hemmsen, M. Otto, and P. Kidmose, “Ear-EEG for sleep assessment: A comparison with actigraphy and PSG,” Sleep Breathing, vol. 25, no. 3, pp. 1693–1705, Sep. 2021. 33219908 10.1007/s11325-020-02248-1

[22] K. B. Mikkelsen, D. B. Villadsen, M. Otto, and P. Kidmose, “Automatic sleep staging using ear-EEG,” Biomed. Eng. OnLine, vol. 16, no. 1, p. 111, Dec. 2017. 28927417 10.1186/s12938-017-0400-5 PMC5606130

[23] D. Looney , “The in-the-ear recording concept: User-centered and wearable brain monitoring,” IEEE Pulse, vol. 3, no. 6, pp. 32–42, Nov. 2012. 23247157 10.1109/MPUL.2012.2216717

[24] E. Occhipinti, H. J. Davies, G. Hammour, and D. P. Mandic, “Hearables: Artefact removal in ear-EEG for continuous 24/7 monitoring,” in Proc. Int. Joint Conf. Neural Netw. (IJCNN), 2022, pp. 1–6.

[25] G. Hammour, M. Yarici, W. V. Rosenberg, and D. P. Mandic, “Hearables: Feasibility and validation of in-ear electrocardiogram,” in Proc. 41st Annu. Int. Conf. IEEE Eng. Med. Biol. Soc. (EMBC), Jul. 2019, pp. 5777–5780. 10.1109/EMBC.2019.8857547 31947165

[26] M. Yarici , “Hearables: Feasibility of recording cardiac rhythms from single ear locations,” 2023, arXiv:2301.02475. 10.1098/rsos.221620 PMC10762432 38179073

[27] G. Hammour and D. P. Mandic, “An in-ear PPG-based blood glucose monitor: A proof-of-concept study,” Sensors, vol. 23, no. 6, p. 3319, Mar. 2023. 36992029 10.3390/s23063319 PMC10057625

[28] H. J. Davies , “In-ear SpO2 for classification of cognitive workload,” IEEE Trans. Cognit. Develop. Syst., vol. 15, no. 2, pp. 950–958, Jun. 2023.

[29] G. M. Hammour and D. P. Mandic, “Hearables: Making sense from motion artefacts in ear-EEG for real-life human activity classification,” in Proc. 43rd Annu. Int. Conf. IEEE Eng. Med. Biol. Soc. (EMBC), Nov. 2021, pp. 6889–6893. 10.1109/EMBC46164.2021.9629886 34892689

[30] A. Supratak and Y. Guo, “TinySleepNet: An efficient deep learning model for sleep stage scoring based on raw single-channel EEG,” in Proc. 42nd Annu. Int. Conf. IEEE Eng. Med. Biol. Soc. (EMBC), Jul. 2020, pp. 641–644. 10.1109/EMBC44109.2020.9176741 33018069

[31] M. Perslev , “U-Sleep: Resilient high-frequency sleep staging,” NPJ Digit. Med., vol. 4, no. 1, p. 72, 2021. 33859353 10.1038/s41746-021-00440-5 PMC8050216

[32] R. Vallat and M. P. Walker, “An open-source, high-performance tool for automated sleep staging,” eLife, vol. 10, Oct. 2021, Art. no. e70092. 10.7554/eLife.70092 PMC8516415 34648426

[33] T. Nakamura, T. Adjei, Y. Alqurashi, D. Looney, M. J. Morrell, and D. P. Mandic, “Complexity science for sleep stage classification from EEG,” in Proc. Int. Joint Conf. Neural Netw. (IJCNN), 2017, pp. 4387–4394.

[34] C. D. Monica , “A protocol for evaluating digital technology for monitoring sleep and circadian rhythms in older people and people living with dementia in the community,” Clocks Sleep, vol. 6, no. 1, pp. 129–155, Feb 2024. 38534798 10.3390/clockssleep6010010 PMC10968838

[35] N. Michielli, U. R. Acharya, and F. Molinari, “Cascaded LSTM recurrent neural network for automated sleep stage classification using single-channel EEG signals,” Comput. Biol. Med., vol. 106, pp. 71–81, Mar. 2019. 30685634 10.1016/j.compbiomed.2019.01.013

[36] G. Ke , “LightGBM: A highly efficient gradient boosting decision tree,” in Proc. Adv. Neural Inf. Process. Syst., vol. 30, 2017, pp. 1–9.

[37] B. Zhai, I. Perez-Pozuelo, E. A. D. Clifton, J. Palotti, and Y. Guan, “Making sense of sleep: Multimodal sleep stage classification in a large, diverse population using movement and cardiac sensing,” Proc. ACM Interact., Mobile, Wearable Ubiquitous Technol., vol. 4, no. 2, pp. 1–33, Jun. 2020.

[38] M. E. Peltola , “Routine and sleep EEG: Minimum recording standards of the international federation of clinical neurophysiology and the international league against epilepsy,” Clin. Neurophysiol., vol. 147, pp. 108–120, Mar. 2023. 36775678 10.1016/j.clinph.2023.01.002

[39] K. Crowley, “Sleep and sleep disorders in older adults,” Neuropsychol. Rev., vol. 21, no. 1, pp. 41–53, Mar. 2011. 21225347 10.1007/s11065-010-9154-6

